# Adaptively Lightweight Spatiotemporal Information-Extraction-Operator-Based DL Method for Aero-Engine RUL Prediction

**DOI:** 10.3390/s23136163

**Published:** 2023-07-05

**Authors:** Junren Shi, Jun Gao, Sheng Xiang

**Affiliations:** 1 School of Automation, Chongqing University of Posts and Telecommunications, Chongqing 400044, China; shijr@cqupt.edu.cn; 2 School of Computer Science and Technology, Chongqing University of Posts and Telecommunications, Chongqing 400044, China; gaojun@cqupt.edu.cn

**Keywords:** RUL prediction, spatiotemporal information, aero-engine, deep learning

## Abstract

Accurate prediction of machine RUL plays a crucial role in reducing human casualties and economic losses, which is of significance. The ability to handle spatiotemporal information contributes to improving the prediction performance of machine RUL. However, most existing models for spatiotemporal information processing are not only complex in structure but also lack adaptive feature extraction capabilities. Therefore, a lightweight operator with adaptive spatiotemporal information extraction ability named Involution GRU (Inv-GRU) is proposed for aero-engine RUL prediction. Involution, the adaptive feature extraction operator, is replaced by the information connection in the gated recurrent unit to achieve adaptively spatiotemporal information extraction and reduce the parameters. Thus, Inv-GRU can well extract the degradation information of the aero-engine. Then, for the RUL prediction task, the Inv-GRU-based deep learning (DL) framework is firstly constructed, where features extracted by Inv-GRU and several human-made features are separately processed to generate health indicators (HIs) from multi-raw data of aero-engines. Finally, fully connected layers are adopted to reduce the dimension and regress RUL based on the generated HIs. By applying the Inv-GRU-based DL framework to the Commercial Modular Aero Propulsion System Simulation (C-MAPSS) datasets, successful predictions of aero-engines RUL have been achieved. Quantitative comparative experiments have demonstrated the advantage of the proposed method over other approaches in terms of both RUL prediction accuracy and computational burden.

## 1. Introduction

Remaining useful life (RUL) prediction, as a significant research domain in prognostics and health management (PHM) [1], offers the potential to forecast the future degradation trajectory of equipment based on its current condition. Transforming scheduled maintenance into proactive operations substantially mitigates the risks of personnel casualties and economic losses resulting from mechanical failures.

With the increasing complexity and sophistication of equipment, conventional PHM methods by dynamic models, expert knowledge, and manual feature extraction have become increasingly limited. Nowadays, fueled by rapid advancements in technologies such as sensors, the Internet of Things, and artificial intelligence, attention has been drawn to DL-based techniques with remarkable performance for RUL prediction [2,3,4]. Therefore, with industrial data accumulation, conducting DL-based RUL prediction research for equipment, which possess powerful feature extraction capabilities, has not only emerged as a hot research topic in academia but also holds significant practical implications for the industry.

DL-based methods enable the construction of deep neural network architectures, endowing them with more powerful feature extraction capabilities compared with shallow machine learning algorithms. Consequently, these methods can directly learn and optimize features from raw data obtained from complex equipment, as well as infer the RUL, thereby enhancing the accuracy and robustness of RUL estimation. Among various DL techniques, neural networks (NNs) have emerged as state-of-the-art models for addressing RUL prediction problems, attracting significant attention from researchers [5,6,7,8].

Recurrent neural networks (RNNs) use the input data and historical data as the final input matrix, which is different from other NNs. Based on this unique design, RNN is well-suited for processing sequential data and has been successfully applied in RUL prediction [9,10]. However, RNN also has its limitations, such as a long recursion time, which indirectly increases the depth and training time of the NN, as well as the frequently occurring issue of vanishing gradients [11]. Long short-term memory (LSTM) was deduced by Hochreiter and Schmidhuber to address the above issues in 1997 [12], and can mitigate the problem of long-term dependencies in RNN and has gained widespread application [13,14].

By comparing the aircraft engines’ prediction performance of the vanilla RNN, LSTM, and gated recurrent unit (GRU), Yuan et al. [15] concluded that LSTM and GRU outperformed traditional RRNs. To solve the degradation problem in deep LSTM models, a residual structure is adopted [16]. Zhao et al. [17] conducted an empirical evaluation of an LSTM-based machine tool wear detection system. They applied the LSTM model to encode raw measurement data into vectors for corresponding tool wear prediction. Wu et al. [18] found that the fusion of multi-sensor inputs can enhance the long-term prediction capability of DLSTM. Guo et al. [19] proposed a novel artificial feature constructed from temporal and frequency domain features to boost the prediction accuracy of LSTM. As a commonly used variant of LSTM, GRU has attracted significant attention thanks to its simplified gating mechanism, which reduces the training burden without compromising the regression capability. Zhao et al. [20] presented a GRU model with local features for machine health monitoring. Zhou et al. [21] introduced an enhanced memory GRU network that utilizes previous state data for predicting bearings’ RUL. He et al. [22] employed a fault-mode-assisted GRU method for RUL prediction to guide the initiation of the predictive maintenance time of machines. Que et al. [23] developed a combined method by stacked GRU, attention mechanism, and Bayesian methods to predict the RUL of bearings. A deep multi-scale feature fusion network based on multi-sensor data to predict the RUL of aircraft engines was proposed by Li et al. [24], with GRU replacing the commonly used fully connected layers for regression prediction. Ni et al. [25] used GRU to predict the RUL of bearing systems and adaptively adjusted the optimal hyper-parameters using a Bayesian optimization algorithm. Zhang et al. [26] proposed a dual-task network structure based on bidirectional GRU and multi-gate expert fusion units, which can simultaneously assess the health condition of aircraft engines and predict their RUL. Ma et al. [27] introduced a novel deep wavelet sequence GRU prediction model to predict the RUL of rotating machinery, where the proposed wavelet sequence GRU generates wavelet sequences at different scales through wavelet layers.

CNN exhibits powerful spatial feature extraction capabilities and is suitable for classification tasks such as fault diagnosis [28]. However, it is rarely adopted alone for RUL prediction. To enhance the model’s ability to extract temporal and spatial information in the RUL prediction task, combining the CNN with RNN or adopting the convolution operators to replace the operations in RNN is the common approach [29,30]. Some researchers combine these two classical models serially and in parallel to construct novel models. Wang et al. [31] replaced the conventional full connections of forward and recurrent processes of GRU with convolutional operators. Similarly, Ma et al. [32] further replaced the full connection on the state-to-state transitions of LSTM as a convolution connection to boost the feature extraction ability. To improve the RUL prediction accuracy, Li et al. [33] presented a combination method by the ConvLSTM and self-attention mechanism. Cheng et al. [34] introduced a new LSTM variant to predict the RUL of aircraft engines by combining autoencoders and RNNs. The proposed method conducted the pooling operation with LSTM’s gating mechanism while retaining the convolutional operations, allowing for parallel processing. Dulaimi et al. [35] proposed a parallel DL framework based on CNN and LSTM to extract the temporal and spatial features from raw measurements. To solve the inconsistent problem of inputs, Xia et al. [36] proposed a CNN-BLSTM method, which has a different time scale processing ability. Xue et al. [37] introduced a data-driven approach for predicting the RUL, which incorporates two parallel pathways: one pathway combines multi-scale CNN and BLSTM, while the other pathway only utilizes BLSTM.

Research works based on LSTM variants and convolution operators have achieved significant success in RUL prediction, but they still have some gaps. The convolutional kernel exhibits redundancy in the channel dimension, and the extraction features lack the ability to adapt flexibly based on the input itself [38]. Moreover, the ability to capture flexible spatiotemporal features not only saves computational resources, but also enables the extraction of rich features, thereby improving the accuracy of mechanical RUL prediction. Additionally, the computation burden is also an important requirement for mechanical RUL prediction. Therefore, it is worth investigating how to enhance the spatiotemporal capturing capability of prediction models while minimizing model parameters to improve prediction speed. 

Consequently, considering the aforementioned limitations, a lightweight operator with adaptive feature capturing capabilities named involution GRU (InvGRU) is proposed, and a deep learning framework is constructed based on this operator to predict the RUL of aircraft engines. The RUL prediction results of the C-MAPSS dataset [24] demonstrate that the proposed method outperforms other publicly available methods in terms of prediction accuracy and computational burden. 

Below are the contributions of the article:

- Introducing InvGRU: We propose a novel operator called InvGRU, which replaces the connection operator in GRU and allows for adaptive capture of spatiotemporal information based on the input itself. InvGRU demonstrates the ability to extract spatiotemporal information with fewer parameters compared with other models.
- Constructing a deep learning framework: Building upon InvGRU, we construct a deep learning framework that achieves higher prediction accuracy.
- Experimental validation: The experimental results on aircraft engine RUL prediction validate the effectiveness and superiority of the proposed InvGRU-based deep learning framework. It outperforms other models in terms of prediction accuracy and showcases the potential for improved RUL estimation in practical applications.

The outline of the article is as follows. Section 1 introduces the research topic. Section 2 presents a concise explanation of the fundamental principles of GRU and involution. In Section 3, the novel operator InvGRU, which has the adaptively spatiotemporal information extraction ability, is introduced. Then, the proposed methods are thoroughly validated and compared through experiments on the C-MAPSS dataset in Section 4. Finally, Section 5 presents the conclusion.

## 2. Theoretical Basis

### 2.1. Inverse Convolution

Thanks to its spatial invariance and channel specificity, CNN has been widely employed for feature extraction. The formula for CNN is as follows:

(1) $$Y_{i,j,k}=\sum_{c=1}^{C_{i}}\sum_{(u,v)\in \Delta K}F_{k,c,u+[K/2],v+[K/2]}X_{i+u,j+v,c}$$

**Figure 1:**
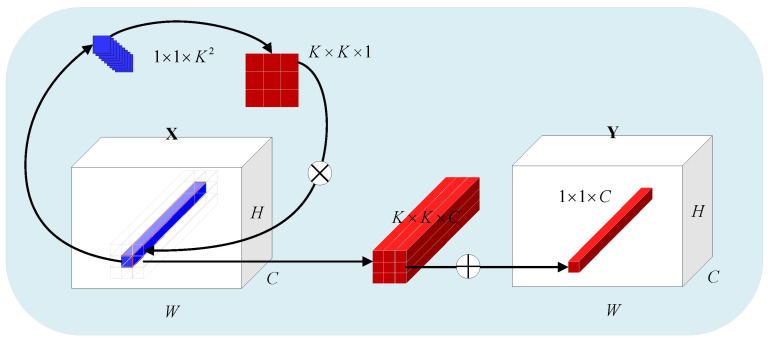
Principle of involution (G = 1).

(2) Δk=[−[K/2],…,[K/2]]×[−[K/2],…,[K/2]]

where $X\in R^{H\times W\times C_{i}}$ and $Y\in R^{H\times W\times C_{o}}$ are the input tensor and the output tensor, respectively; $F\in R^{C_{o}\times C_{i}\times K\times K}$ denotes the kernel of convolution; $c_{o}$, $c_{i}$, and *K* denote the output channel number, input channel number, and kernel size, respectively; while *H* and *W* represent the spatial dimensions of the output and input channels. Although sharing spatial parameters alleviates some computational burden, it also introduces certain drawbacks. For instance, the extracted features tend to be relatively simplistic, and the convolution kernel lacks flexibility in adapting to input data [38]. Furthermore, the convolutional kernel exhibits redundancy in the channel dimension [38]. The recently proposed inverse convolutional neural network (INN) [38] addresses the aforementioned limitations in a manner that preserves channel invariance and spatial specificity. For the channel dimension, INN allows for sharing of involution kernels, causing INN to provide more flexible modeling of the involution kernels in the spatial dimension, thereby exhibiting characteristics opposite to those of convolutional neural networks. The mathematical expression of INN is as follows:

(3) $$Y_{i,j,k}=\sum_{(u,v)\in \Delta K}H_{i,j,u+[K/2],v+[K/2],[kG/C]}X_{i+u,j+v,k}$$

where $H\in R^{H\times W\times K\times K\times G}$ represents the kernel of involution, *G* represents that all of the channels share *G* involution kernels, and it is noted that G≪C. Compared with CNN, INN cannot utilize fixed weight matrices as learnable parameters. Instead, it generates corresponding involution kernels by the input features.

(4) $$H_{i,j}=\Phi (X_{\Psi_{i,j}})=W_{1}\mathrm{Relu}\left(\mathrm{BN}\left(W_{0}X_{i,j}\right)\right)$$

where $W_{0}\in R^{\frac{c}{r}\times c}$ and $W_{1}\in R^{(K\times K\times G)\times \frac{c}{r}}$ denote the linear transformation matrix, *r* is the channel reduction rate, BN is the batch normalization, Relu is the Relu activation function, and $X_{\Psi_{i,j}}$ denotes the index set of coordinate (*i*, *j*). The principle of INN is shown in Figure 1, which is demonstrated as the example when *G* = 1.

### 2.2. GRU

**Figure 2:**
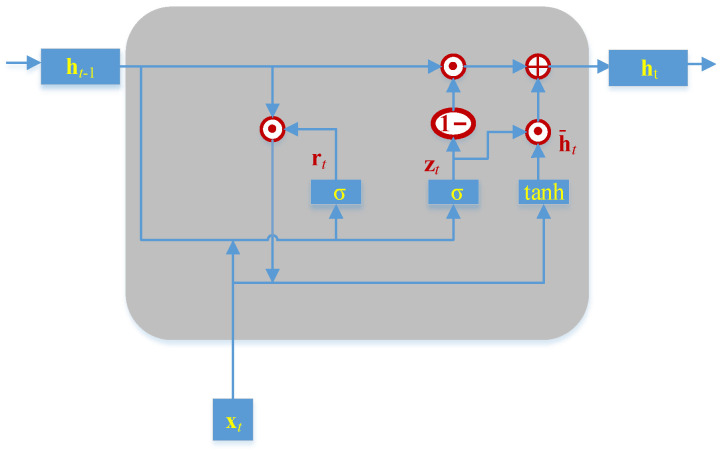
Schematic diagram of GRU, in which ⊕ is the addition operator.

GRU, which had fewer parameters compared with LSTM, only has the reset gate $r_{t}$ and an update gate $z_{t}$. The structure of GRU is demonstrated in Figure 2. The output $h_{t}$ of GRU at the current time step t can be represented by the following equation:

(5) $$\begin{array}{l} z_{t}=\sigma \left(w_{zx}x_{t}+w_{zh}h_{t-1}+b_{z}\right)\\ r_{t}=\sigma \left(w_{rx}x_{t}+w_{rh}h_{t-1}+b_{r}\right)\\ {\bar{h}}_{t}=\tanh\left(u_{h}x_{t}+w_{h}\left(r_{t}⊙h_{t-1}\right)+b_{h}\right)\\ h_{t}=\left(1-z_{t}\right)⊙h_{t-1}+z_{t}⊙{\bar{h}}_{t} \end{array}$$

where w denotes the weight matrix of the input data $x_{t}$ and recurrent data $h_{t-1}$; b is the bias; ${\bar{h}}_{t}$ represents the hidden state; ⊙ is the dot product operator; tanh and σ are the activation functions; and $h_{t}$ denotes the output data.

## 3. Proposed Methodology

### 3.1. Proposed InvGRU

**Figure 3:**
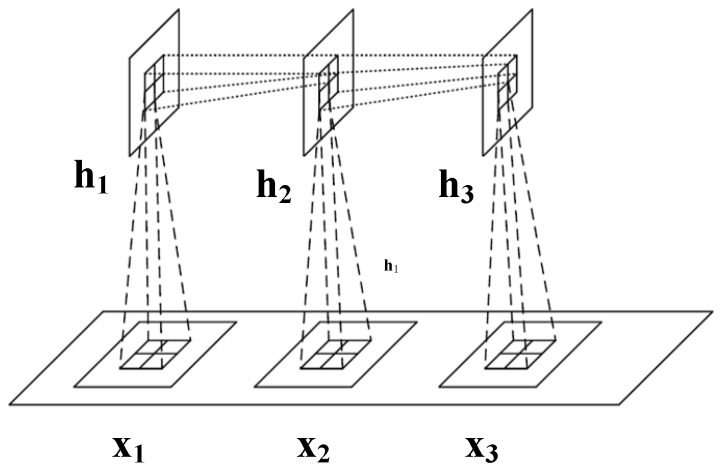
Schematic diagram of InvGRU, where $X_{1},X_{2},X_{3}$ are the time series input matrix and $h_{1},h_{2},h_{3}$ are the hidden features extracted by the involution operator.

Using convolutional operations to learn representations from multi-source raw data has been shown to outperform hand-crafted features in machine diagnosis and prognosis [28,29,30]. Recent studies have proposed combining RNN models with CNN representations to capture spatio-temporal information [35,36,37]. This approach improves the model’s ability to understand patterns and relationships over space and time, leading to better analysis and prediction in various domains. A novel operator called Involution GRU (InvGRU) is proposed to address the limitations of the convolution operator. InvGRU introduces involution operations in both the input-to-state and state-to-state transitions, enabling adaptive feature extraction from multi-source raw data while reducing model parameters. This approach enhances the model’s ability to capture spatio-temporal information effectively. The diagram of InvGRU is shown in Figure 3.

To enhance the feature processing method for one-dimensional time series data, a one-dimensional involution algorithm based on one-dimensional vectors as inputs, namely 1D-INN, is adopted. The mathematical expression of 1D-INN is presented below:

(6) $$Y_{i,k}=\sum_{u\in \Delta K}H_{i,u+[K/2],[kG/C]}X_{i+u,k}$$

(7) $$H_{i}=\Phi (X_{\Psi_{i}})=W_{1}\mathrm{Mish}\left(\mathrm{BN}\left(W_{0}X_{i}\right)\right)$$

where $H\in R^{H\times K\times G}$ is the kernel of ID-INN, $X_{\Psi_{i}}$ is the index set of coordinate (*i*, 1), $W_{0}\in R^{\frac{c}{r}\times c}$ and $W_{1}\in R^{(K\times G)\times \frac{c}{r}}$ are the weight connection matrixes to make a linear transformation, and Mish is the Mish activation function. The other parameters are the same as raw INN. We enhance the feature representation using INN to incorporate longer temporal convolutions, allowing for the prediction of RUL at a larger temporal scale. In the article, the INN kernel is set to 5 and the size of max-pooling is set to 2, while *r* is set to 2. InvGRU, similar to the conventional GRU, comprises update gates, reset gates, and cells. The forward process of InvGRU, responsible for computing the output, is defined by the following equations:

Update gates:

(8) $$A_{z}^{t}=w_{zx}*x_{t}+w_{zh}*h_{t-1}+b_{z}$$

(9) $$z_{t}=\sigma \left(A_{z}^{t}\right)$$

Reset gates:

(10) $$A_{r}^{t}=w_{rx}*x_{t}+w_{rh}*h_{t-1}+b_{r}$$

(11) $$r_{t}=\sigma \left(A_{r}^{t}\right)$$

Cells:

(12) $$A_{h}^{t}=u_{h}*x_{t}+w_{h}*\left(r_{t}⊙h_{t-1}\right)+b_{h}$$

(13) $${\bar{h}}_{t}=\tanh\left(A_{h}^{t}\right)$$

Cell outputs:

(14) $$h_{t}=\left(1-z_{t}\right)⊙h_{t-1}+z_{t}⊙{\bar{h}}_{t}$$

where ∗ is the operator of the ID-INN; w and b terms are the learnable weights and biases, respectively; and the other parameters are the same as GRU.

### 3.2. The Adopted DL Framework

**Figure 4:**
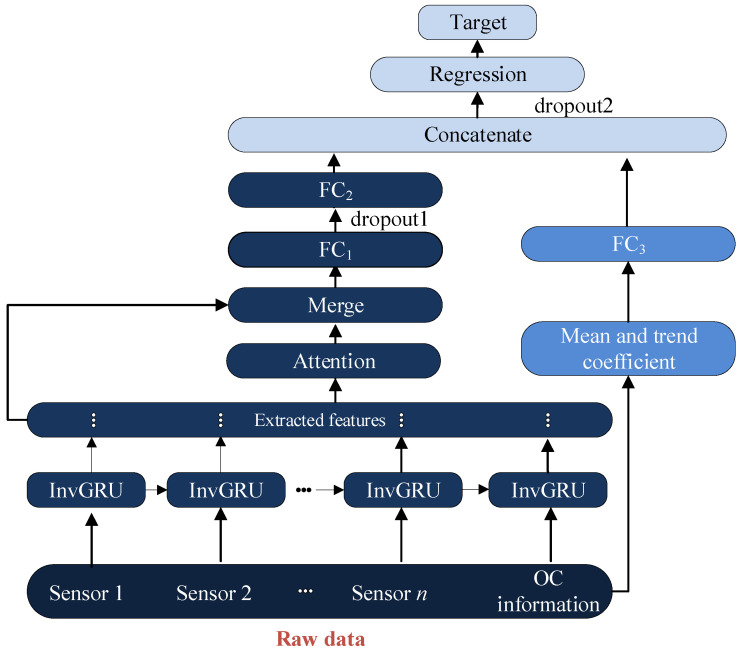
InvGRU-based DL framework.

Based on the proposed InvGRU, a DL framework is adopted to estimate the aero-engines’ RUL. The framework diagram in Figure 4 integrates HIs from both neural networks (NNs) and human-made features, enabling a comprehensive approach to RUL prediction. First, InvGRU is employed to extract features based on multi-raw measurements, including multiple sensors’ data and engine operational condition (OC) information. The attention weights [39] are calculated by the obtained hidden features and combined with the hidden features, and the merged features are input into the following FC layers to generate the HIs from the NN. In the next step, commonly used handcrafted features such as the mean and trend coefficient are calculated from the raw data. The mean represents the average value of a window, while the trend coefficient corresponds to the slope coefficient derived from linear regression on the windowed time series. To obtain the HIs of human-made features, these handcrafted features are then fed into a new fully connected FC layer. Finally, HIs obtained from the neural network and human-made features are concatenated to form the HI set. This concatenated set is inputted into the regression layer, which predicts the RUL.

During the training, supposed that *N* represents the sample number and the loss function is the mean square error (MSE), which is adopted to evaluate the similarity between the predicted RUL ${\bar{Rul}}_{i}$ and the true RUL $Rul_{i}$ of each sample *i*. The MSE is calculated using Equation (15) as follows:

(15) $$L\left(MSE,\theta \right)=\frac{1}{2}\sum_{i=1}^{N}{\left({\bar{Rul}}_{i}-Rul_{i}\right)}^{2}$$

Adam is used as the optimization method to tune the parameters θ of the proposed method based on the error gradients during the back-propagation processing. Dropout, a technique for preventing overfitting, is implemented in the model during training. Table 1 shows the hyper-parameters of the prosed DL framework based on InvGRU.

## 4. Experimental Analysis

### 4.1. Evaluation Indexes

The RUL prediction performance of the method is quantitatively characterized using score and root mean square error (RMSE), which are defined by the following formulas:

(16) $$A_{i}=\left\{\begin{array}{cc} exp(-((\bar{Rul_{i}}-Rul_{i})/13))-1, & \bar{Rul_{i}}<Rul_{i}\\ exp((\bar{Rul_{i}}-Rul_{i})/10)-1, & \bar{Rul_{i}}\geq Rul_{i} \end{array}\right.$$

(17) $$Score=\sum_{i=1}^{N}A_{i}$$

(18) $$RMSE=\sqrt{\frac{1}{N}\sum_{i=1}^{N}{\left(Rul_{i}-\bar{Rul_{i}}\right)}^{2}}$$

**Figure 5:**
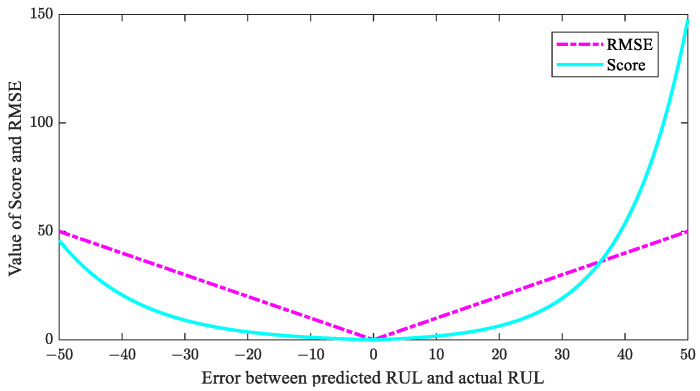
The curves of the two evaluation indexes.

These metric values are inversely proportional to the RUL prediction performance. In other words, a lower value indicates better model performance. Score penalizes delayed predictions more heavily than RMSE, as shown in Figure 5, making it more aligned with engineering practices. Therefore, the score is more reasonable, especially when the RMSE values are close. In the figure, the vertical axis represents the value of RMSE and score, while the horizontal axis represents the errors between the predicted RUL and actual RUL.

### 4.2. The Details of the C-MAPSS Dataset

**Figure 6:**
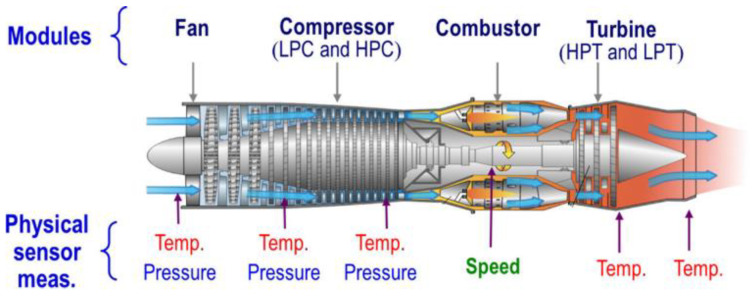
Diagram of the aircraft engine.

The C-MAPSS dataset, developed by NASA, simulates degradation data for turbofan engines, whose structure is shown in Figure 6. The C-MAPSS dataset can be divided into four subsets based on different operating conditions and fault modes, as described in Table 2. The 21 simulation outputs of C-MAPSS are listed in Table 3, in which ~, ↑ and ↓ represent the stable, upward and descend trends of the sensor measurements.

Each subset of the dataset consists of training data, testing data, and corresponding actual RUL values. The training data comprise all engine data from a healthy state to failure, while the testing data include data from engines that were operated before failure. In both the training and testing datasets, a diverse set of engines with varying initial health states is included. This results in variations in the operating cycles of different engines within the same dataset, reflecting the heterogeneous nature of the engine population. To demonstrate the effectiveness of the proposed method, experiments are conducted on all subsets of the dataset.

### 4.3. Data Preprocessing

**Figure 7:**
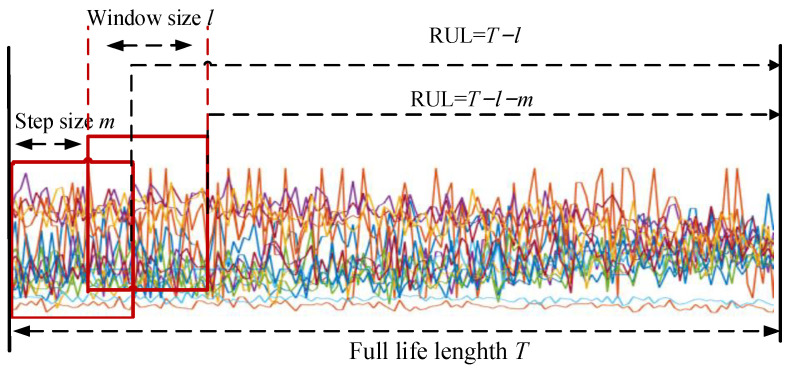
Processing of data segmentation.

Firstly, not all sensor measurements are included as inputs in the RUL prediction model. Some stable measurements (sensors 1, 5, 6, 10, 16, 18, and 19) are excluded in advance. These sensor measurements contain limited degradation information of the engine and are not suitable for predicting the RUL. Additionally, operating condition information affects the predictive capability of the model. Therefore, the 14 selected sensor measurements and operating condition information serve as the final input for the model. Secondly, we segment the data using the technique demonstrated in Figure 7. *T*, *l*, and *m* represent the total lifecycle, the window size, and the sliding step, respectively. The size of the *i*-th input is *l* × *n*, where *n* represents the dimension number of the final input of the proposed model. The RUL at this point is *T*s − *l* − (*i* − 1) × *m*. Based on the results of the experiments, the sliding window size *l* is set to 30 and the sliding step *m* is set to 1. Finally, the linear piecewise RUL technique is used to construct the RUL labels as follows:

(19) $$Rul=\left\{\begin{array}{cc} Rul, & if\;Rul\leq Rul_{\max}\\ Rul_{\max}, & if\;Rul>Rul_{\max} \end{array}\right.$$

where the preset $Rul_{\max}$ is 125.

### 4.4. The Analysis and Comparison of RUL Prediction Results

**Figure 8:**
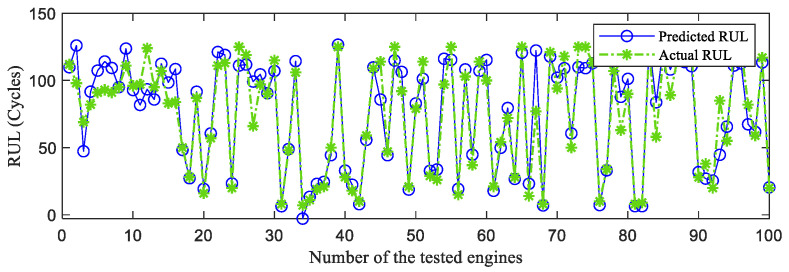
RUL prediction performance on FD001.

**Figure 9:**
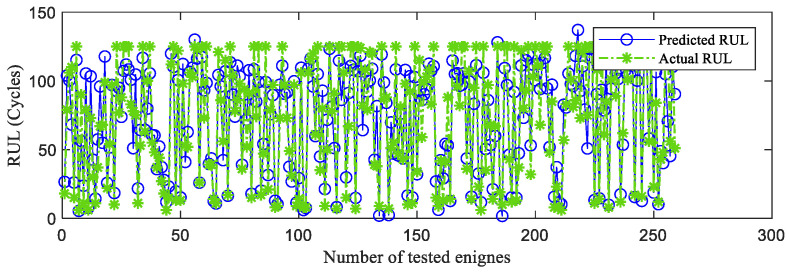
RUL prediction performance on FD002.

**Figure 10:**
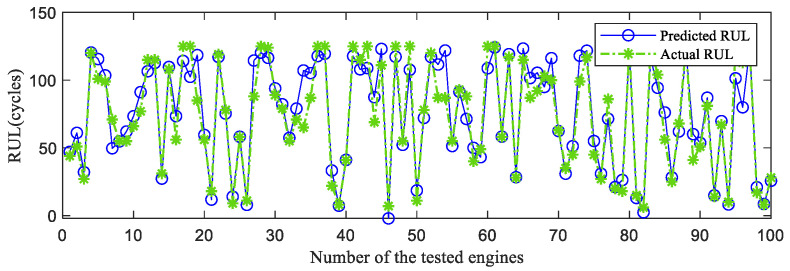
RUL prediction performance on FD003.

**Figure 11:**
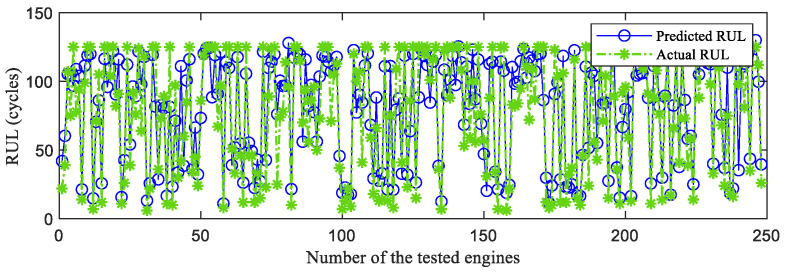
RUL prediction performance on FD004.

**Figure 12:**
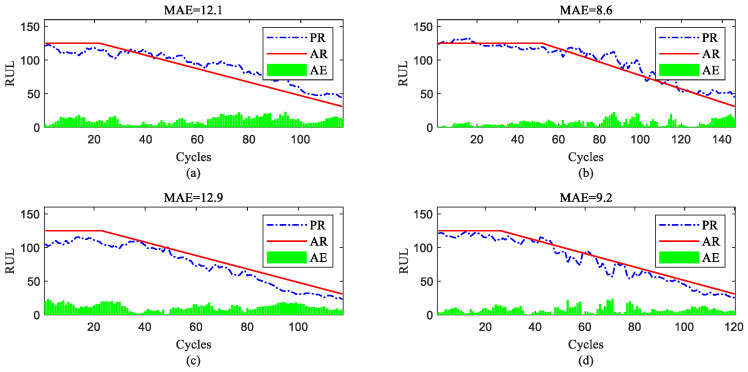
RUL prediction performance of engines of FD001 ((**a**) engine #46, (**b**) engine #58, (**c**) engine #66, and (**d**) engine #92).

First, the proposed InvGRU-based DL framework is trained using the training sets from all of the subsets. Then, the test set of the subsets is adopted to test the predictive performance of InvGRU-based DL framework. The prediction results are shown in Figure 8, Figure 9, Figure 10, Figure 11 and Figure 12. In the figures, the x-axis is the tested aircraft engine unit number and the y-axis denotes the RUL cycles. The predicted RUL and the actual RUL are represented by the solid blue line and dashed green line, respectively.

From Figure 8, Figure 9, Figure 10, Figure 11 and Figure 12, it can be observed that, across all subsets (FD001, FD002, FD003, and FD004), the proposed model demonstrates a consistent prediction of the RUL that aligns closely with the actual RUL for the majority of the tested aircraft engine units. This is evident from the substantial overlap between the blue and green data points, indicating the high accuracy of the proposed model in predicting RUL. Upon closer examination, Figure 8 shows a closer proximity between the RUL and the actual RUL compared with Figure 9, Figure 10 and Figure 11. This indicates that the proposed model achieves its best performance on the FD001 dataset. Additionally, the RUL prediction performance of the proposed method is superior on the FD003 dataset compared with the FD002 dataset, while it performs worst on the FD004 dataset. Moreover, the RUL prediction effectiveness of the proposed model is higher on the FD001 and FD003 datasets compared with the FD002 and FD004 datasets, highlighting its superior performance under consistent failure modes (FD001 and FD003) compared with multiple operating conditions (FD002 and FD004). This is attributed to the relatively simpler degradation trend of engines under a single operating condition, coupled with significant overlap between the training and testing sets. Furthermore, the accuracy of RUL prediction results is higher for the FD001 dataset than for the FD003 dataset, and higher for the FD002 dataset than for the FD004 dataset. This suggests that, under consistent operating conditions, the proposed model exhibits better RUL prediction performance for single failure modes (FD001 and FD002) compared with composite failure modes (FD003 and FD004). Hence, the proposed model demonstrates higher RUL prediction accuracy for single failure modes compared with multiple failure modes. Additionally, the RUL prediction results on the FD003 dataset surpass those on the FD002 dataset, indicating that the complex failure mode in the C-MAPSS dataset has less influence on the RUL prediction of the proposed model compared with the operating conditions of the aircraft engine units. 

**Figure 13:**
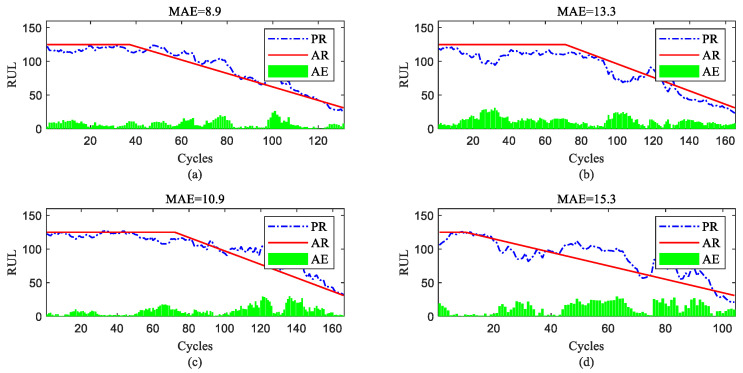
RUL prediction performance of engines of FD002 ((**a**) engine #9, (**b**) engine #45, (**c**) engine #150, and (**d**) engine #182).

**Figure 14:**
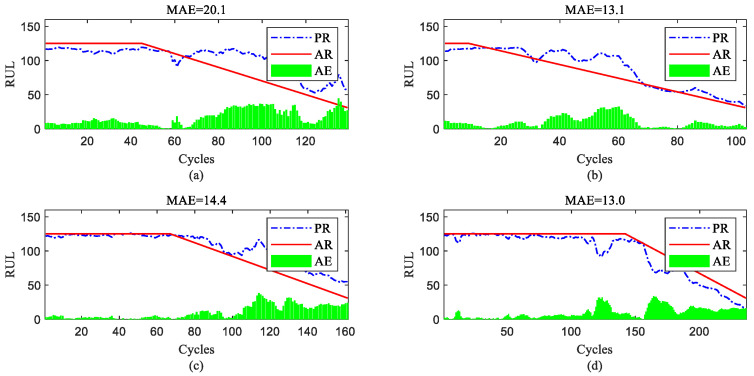
RUL prediction performance of engines of FD003 ((**a**) engine #25, (**b**) engine #38, (**c**) engine #75, and (**d**) engine #92).

**Figure 15:**
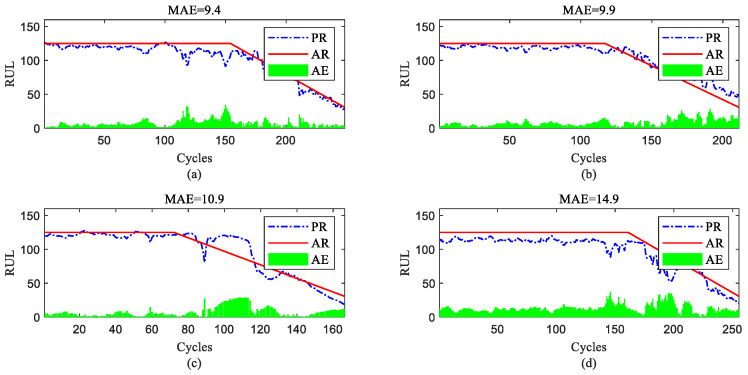
RUL prediction performance of engines of FD004 ((**a**) engine #35, (**b**) engine #68, (**c**) engine #100, and (**d**) engine #151).

To further show the InvGRU-based DL framework performance in predicting the RUL of individual engine units during the overall degradation process, four test engine units randomly selected from all subsets were used to showcase the full-life estimation process shown in Figure 12, Figure 13, Figure 14 and Figure 15. The blue line in the figures represents the predicted RUL (PR) of the engine unit, while the red line represents the actual RUL (AR). The green bars represent the absolute error (AE) between PR and AR for each cycle. Additionally, the mean of the absolute errors (MAE) between PR and AR across all cycles of the engine unit was computed to evaluate the average prediction error.

It can be observed from Figure 12, Figure 13, Figure 14 and Figure 15 that the predicted RUL of the selected test engine units closely aligns with the actual RUL, effectively revealing their degradation trends. Moreover, considering the average values of the MAE in Figure 12, Figure 13, Figure 14 and Figure 15, the average MAE on the FD001 dataset is 10.7, while the average MAE on the FD002, FD003, and FD004 datasets are 12.1, 15.2, and 11.3, respectively. This indicates that the proposed model exhibits significantly better RUL prediction performance on the FD001 dataset compared with the FD002, FD003, and FD004 datasets. As the number of engine cycles gradually increases, the degradation process begins to manifest and worsen. For most engines, the accuracy of predicting the RUL in the later stages of the degradation process tends to be higher than in the earlier stages. This is evident in Figure 12c, Figure 13a–c, Figure 14b,d and Figure 15a,c,d.

To demonstrate the lightweight nature of the proposed methods and illustrate the lower computational resource consumption, we compare the parameter count and computational cost of the models. For general validation purposes, INN and CNN are employed in a two-dimensional configuration. The parameter count of INN is $\frac{K^{2}GC+C^{2}}{r}$, while the computational burden of INN can be divided into two parts: the involution kernel generation component, which is $HW\times \frac{K^{2}GC+C^{2}}{r}$, and the multiplication-addition component, which is $HW\times K^{2}C$. On the other hand, CNN has a parameter count and computational burden of $K^{2}C^{2}$ and $HW\times K^{2}C^{2}$, respectively, which is higher than that of INN. This indicates that, under the same hyper-parameters, INN has a smaller computational load compared with CNN. Simultaneously, GRU has a parameter count of $9\times c_{n}$ and a computational burden of $8\times (c_{n}\times i_{l})+3\times {\left(c_{n}\times i_{l}\right)}^{2}+3\times ({c_{n}}^{3}\times {i_{l}}^{2})+9\times ({c_{n}}^{3}\times {i_{l}}^{4})$, while LSTM has a parameter count of $12\times c_{n}$ and a computational burden of $12\times (c_{n}\times i_{l})+18\times {\left(c_{n}\times i_{l}\right)}^{2}+54\times {\left(c_{n}\times i_{l}\right)}^{3}$, where $c_{n}$ represents the number of hidden neurons and $i_{l}$ represents the input length. GRU exhibits lower computational costs compared with LSTM. From this observation, it is evident that the computational complexity of InvGRU is lower than that of ConvLSTM.

To evaluate the computational efficiency, we selected the challenging FD004 dataset for performance testing. Specifically, we compared the runtime of InvGRU with ConvLSTM on the FD004 dataset. Using the same computing device consisting of Nvidia GeForce RTX2060, Intel(R) Core(TM) i7-10875H, and 16 GB RAM, InvGRU achieved a remarkable 16% reduction in time per epoch, taking only 4 s. In the training stage, each epoch required 8 s and a total of 32 epochs were executed, resulting in a cumulative training time of 256 s. In the testing stage, the inference time was exceptionally fast, with a calculation time of just 0.07 s per sample. Therefore, the proposed method is more concise.

To further highlight the advantages of the InvGRU-based DL framework in predicting RUL, this study conducted comparative experiments on RUL prediction capabilities between the proposed model and several other models, including statistical-based models [34], shallow machine learning models [39], classical deep models [40,41,42], and recently published deep learning models [4,14,34,43]. To obtain comprehensive performance results, these models were subjected to 10 parallel experiments for RUL prediction on each subset. Subsequently, performance evaluation metrics, namely score and RMSE values, were computed based on the prediction results and are presented in Table 4, Table 5 and Table 6. Table 4 displays the evaluation metric values for the compared methods on the FD001 and FD002 datasets, Table 5 presents the evaluation metric values for the compared methods on the FD003 and FD004 datasets, and Table 6 represents the mean evaluation metric values for the compared methods across all subsets, providing an average performance assessment of the predictive capabilities of the compared methods on the C-MAPSS dataset.

Moreover, from Table 4, Table 5 and Table 6, it can be observed that the proposed model exhibits favorable predictive performance and a significant improvement compared with other deep learning models. This demonstrates that the utilization of spatiotemporal information of the input leads to feature diversification and enhances the model’s RUL predictive capability. The proposed Inv-GRU adopted an involution operator to replace the information connection in the gated recurrent unit, enabling the adaptively spatiotemporal information extraction ability and reducing the parameters, and further enhancing the prediction performance of aircraft engine RUL. Based on the aforementioned analysis, it can be concluded that the proposed model exhibits satisfactory universality and accuracy in predicting RUL on the C-MAPSS dataset. Thus, the proposed method can be successfully applied in the aero-engine RUL prediction tasks.

## 5. Conclusions

To overcome the complexity and limited feature extraction capability of conventional models used for processing spatiotemporal information, a lightweight operator called InvGRU is introduced to enhance the prediction of RUL for aero-engines. InvGRU replaces the information connection in the gated recurrent unit with an adaptive feature extraction operator known as Involution. By doing so, InvGRU can dynamically extract spatiotemporal information while reducing the number of parameters involved. The output of InvGRU is then passed through a neural network (NN) to transform it into aero-engine health features. These health features, along with manually crafted features, are concatenated and fed into FC layers for dimension reduction and subsequent RUL estimation. The proposed model is trained using existing data and, once trained, it can be utilized to estimate the RUL of aero-engines using new measurements. The proposed method exhibits a 23.44% improvement in the score metric and an 11.58% improvement in the RMSE metric compared with other methods, highlighting its superiority. These results demonstrate the advantages of the proposed approach in accurately predicting the RUL of aero-engines.

## Figures and Tables

**Table 1:** The hyper-parameters of the prosed DL framework based on InvGRU.

Sub Layer	Hyperparameter Value	Sub Layer	Hyperparameter Value
InvGRU	70	Regression (Linear)	1
FC1 (Relu)	30	Learning rate	0.005
FC2 (Relu)	30	Dropout1	0.5
FC3 (Relu)	10	Dropout2	0.3

**Table 2:** The details of dataset C-MAPSS.

Subset	FD001	FD002	FD003	FD004
Total number of engines	100	260	100	249
Operating condition	1	6	1	6
Type of fault	1	1	2	2
Maximum cycles	362	378	525	543
Minimum cycles	128	128	145	128

**Table 3:** Sensors of C-MAPSS.

Number	Symbol	Description	Unit	Trend	Number	Symbol	Description	Unit	Trend
1	T2	Total fan inlet temperature	ºR	~	12	Phi	Fuel flow ratio to Ps30	pps/psi	↓
2	T24	Total exit temperature of LPC	ºR	↑	13	NRf	Corrected fan speed	rpm	↑
3	T30	HPC total outlet temperature	ºR	↑	14	NRc	Modified core velocity	rpm	↓
4	T50	Total LPT outlet temperature	ºR	↑	15	BPR	Bypass ratio	--	↑
5	P2	Fan inlet pressure	psia	~	16	farB	Burner gas ratio	--	~
6	P15	Total pressure of culvert pipe	psia	~	17	htBleed	Exhaust enthalpy	--	↑
7	P30	Total outlet pressure of HPC	psia	↓	18	NF_dmd	Required fan speed	rpm	~
8	Nf	Physical fan speed	rpm	↑	19	PCNR_dmd	Modify required fan speed	rpm	~
9	Nc	Physical core velocity	rpm	↑	20	W31	HPT coolant flow rate	lbm/s	↓
10	Epr	Engine pressure ratio	--	~	21	W32	LPT coolant flow rate	lbm/s	↓
11	Ps30	HPC outlet static pressure	psia	↑					

**Table 4:** The RUL prediction comparisons of different methods on subset FD001 and FD002.

Model	FD001		FD002	
	Score	RMSE	Score	RMSE
Cox’s regression [34]	28,616	45.10	N/A	N/A
SVR [39]	1382	20.96	58,990	41.99
RVR [39]	1503	23.86	17,423	31.29
RF [39]	480	17.91	70,456	29.59
CNN [40]	1287	18.45	17,423	30.29
LSTM [42]	338	16.14	4450	24.49
DBN [41]	418	15.21	9032	27.12
MONBNE [41]	334	15.04	5590	25.05
LSTM+attention+ handscraft feature [20]	322	14.53	N/A	N/A
Acyclic Graph Network [43]	229	11.96	2730	20.34
AEQRNN [34]	N/A	N/A	3220	19.10
MCLSTM-based [4]	260	13.21	1354	19.82
SMDN [14]	240	13.72	1464	16.77
**Proposed**	**238**	**12.34**	**1205**	**15.59**

**Table 5:** The RUL prediction comparisons of different methods on subset FD003 and FD004.

Model	FD003		FD004	
	Score	RMSE	Score	RMSE
Cox’s regression [34]	N/A	N/A	1,164,590	54.29
SVR [39]	1598	21.04	371,140	45.35
RVR [39]	17,423	22.36	26,509	34.34
RF [39]	711	20.27	46,568	31.12
CNN [40]	1431	19.81	7886	29.16
LSTM [42]	852	16.18	5550	28.17
DBN [41]	442	14.71	7955	29.88
MONBNE [41]	422	12.51	6558	28.66
LSTM+attention+ handscraft feature [20]	N/A	N/A	5649	27.08
Acyclic Graph Network [43]	535	12.46	3370	22.43
AEQRNN [34]	N/A	N/A	4597	20.60
MCLSTM-based [4]	327	13.45	2926	22.10
SMDN [14]	305	12.70	1591	18.24
**Proposed**	**292**	**13.12**	**1020**	**13.25**

**Table 6:** The comparisons of different methods for RUL prediction based on the C-MAPSS dataset.

Model	Mean Performance	
	RMSE	Score
Cox’s regression [34]	49.70	596,603
SVR [39]	32.335	108,277
RVR [39]	27.96	11,716
RF [39]	24.72	29,553
CNN [40]	24.42	7006
LSTM [42]	21.25	2797
DBN [41]	21.73	4461
MONBNE [41]	20.32	3225
LSTM+attention+ handscraft feature [20]	20.80	2985
Acyclic Graph Network [43]	16.80	1716
AEQRNN [34]	19.85	3908
MCLSTM-based [4]	17.40	1216
SMDN [14]	15.36	900
**Proposed**	**13.58**	**689**

## Data Availability

https://data.nasa.gov/dataset/C-MAPSS-Aircraft-Engine-Simulator-Data/xaut-bemq.
